# Rare Recurrent Variants in Noncoding Regions Impact Attention-Deficit Hyperactivity Disorder (ADHD) Gene Networks in Children of both African American and European American Ancestry

**DOI:** 10.3390/genes12020310

**Published:** 2021-02-22

**Authors:** Yichuan Liu, Xiao Chang, Hui-Qi Qu, Lifeng Tian, Joseph Glessner, Jingchun Qu, Dong Li, Haijun Qiu, Patrick Sleiman, Hakon Hakonarson

**Affiliations:** 1 Center for Applied Genomics, Children’s Hospital of Philadelphia, Philadelphia, PA 19104, USA; liuy5@email.chop.edu (Y.L.); changx@email.chop.edu (X.C.); quh@email.chop.edu (H.-Q.Q.); tianl@email.chop.edu (L.T.); glessner@email.chop.edu (J.G.); jingchun.qu789@gmail.com (J.Q.); lid2@email.chop.edu (D.L.); qiuh@email.chop.edu (H.Q.); sleimanp@email.chop.edu (P.S.); 2 Division of Human Genetics, Department of Pediatrics, The Perelman School of Medicine, University of Pennsylvania, Philadelphia, PA 19104, USA; 3 Department of Human Genetics, Children’s Hospital of Philadelphia, Philadelphia, PA 19104, USA

**Keywords:** attention-deficit hyperactivity disorder, whole genome sequence, noncoding regions, genetic variants

## Abstract

Attention-deficit hyperactivity disorder (ADHD) is a neurodevelopmental disorder with poorly understood molecular mechanisms that results in significant impairment in children. In this study, we sought to assess the role of rare recurrent variants in non-European populations and outside of coding regions. We generated whole genome sequence (WGS) data on 875 individuals, including 205 ADHD cases and 670 non-ADHD controls. The cases included 116 African Americans (AA) and 89 European Americans (EA), and the controls included 408 AA and 262 EA. Multiple novel rare recurrent variants were identified in exonic regions, functionally classified as stop-gains and frameshifts for known ADHD genes. Deletion in introns of the protocadherins families and the ncRNA *HGB8P* were identified in two independent EA ADHD patients. A meta-analysis of the two ethnicities for differential ADHD recurrent variants compared to controls shows a small number of overlaps. These results suggest that rare recurrent variants in noncoding regions may be involved in the pathogenesis of ADHD in children of both AA and EA ancestry; thus, WGS could be a powerful discovery tool for studying the molecular mechanisms of ADHD.

## 1. Introduction

Attention-deficit hyperactivity disorder (ADHD) is a common neurological disorder resulting in significant life-long impairments [1]. Previous studies suggest that genomic variation, such as single-nucleotide variants (SNVs), residing in genes that form biological networks that are involved with neurodevelopmental processes, may have significant impact on the ADHD phenotype [2]. Studies have attempted to investigate the genetic susceptibility of ADHD through genome-wide association analysis (GWAS) [3,4]; however the current understanding of the molecular mechanisms underlying this complex disease is incomplete and attempts to replicate previous studies have been inconsistent. Previous studies were highly focused on the coding regions of the genome, and no systematic genetic studies have been conducted in ADHD patients of ethnicities other than European Caucasian. To address these limitations of previous studies, we performed deep whole genome sequencing (WGS) in 875 individuals, including 205 ADHD patients and 670 non-ADHD controls, in order to assess the impact of variants in noncoding regions on the molecular pathways of ADHD. We included a significant number of African Americans in the study, including 116 cases and 408 controls, to expand the analysis into other populations besides Europeans. The results suggest that noncoding variants, especially intronic indels, may play critical roles in the molecular mechanisms underlying ADHD and that population specific variants are present. These results provide useful information and direction for future ADHD studies addressing gene network regulation of new innovative therapies.

## 2. Materials and Methods

### 2.1. Patient’s Information and Whole Genome Sequencing

The patients were recruited by the Center for Applied Genomics (CAG) at The Children’s Hospital of Philadelphia (CHOP), which has established the world’s largest pediatric biobank with full electronic medical record (EMR) coupling. There were 205 ADHD cases, including 116 African Americans (AA, including 42 females and 74 males) and 89 European Americans (EA, including 29 females and 60 males), and 670 controls, including 408 AA and 262 EA, whose samples were whole genome sequenced. The AA cases included 27 patients of attention deficit disorder without hyperactivity and 89 patients of attention deficit disorder with hyperactivity. The EA cases included 34 patients of attention deficit disorder without hyperactivity and 55 patients of attention deficit disorder with hyperactivity. The data is available at the database of Genotypes and Phenotypes (dbGaP) portal with the accession number phs001165.

### 2.2. Whole Genome Sequencing Data Processing

The whole genome sequencing (WGS) using the Illumina platform and average depth coverage is 30× for the genome region. Each lane or sub-lane of data was aligned to GRCh37-lite with bwa v0.7.10 [5]. The unaligned BAMs were converted to interleaved FASTQ format using BEDTools 2.17.0 [6]. Default settings were used for bwa mem with the exception that eight threads were utilized. ReadGroup entries were added to resulting SAM files using gmt sam add-read-group-tag. This SAM file was then converted to a BAM file using Samtools v0.1.19 [7], name sorted (samtools sort -n), mate pairing-assigned (samtools fixmate), re-sorted by position (samtools sort), and indexed using gmt sam index-bam. Read duplication marking and merging reads from multiple lanes, with subsequent merging of the sequencing library as necessary, using Picard v1.113. MergeSamFiles and duplicates were then marked per library using Picard MarkDuplicates v1.113. Lastly, each per-library BAM with duplicates marked was merged together to generate a single BAM file for the sample. For MergeSamFiles, we ran SORT_ORDER=coordinate and MERGE_SEQUENCE_DICTIONARIES=true. For both tools, ASSUME_SORTED=true and VALIDATION_STRINGENCY=SILENT were specified. All other parameters were set to default settings. Samtools flagstat was run on each BAM file generated (per-lane, per-library, and final merged).

### 2.3. Variants Detection

A total of 875 WGS samples were processed and annotated by Illumina Edico Genome’s DRAGEN (Dynamic Read Analysis for Genomics) platform. The DRAGEN platform uses a field-programmable gate array (FPGA) to provide hardware-accelerated implementations of genome pipeline algorithms, including BCL conversion, compression, mapping, alignment, sorting, duplicate marking, and haplotype-based variant calling. ANNOVAR [8] was applied to annotate the variants, the low-confidence and repeated regions were removed, and the occurrences of each variant was counted for ADHD individuals and controls with the annotated outputs from DRAGEN. The final list of variants were reviewed by the Integrative Genomics Viewer (IGV) [9], and the variant calls in low confidence regions were removed.

### 2.4. Rare Recurrent Variants Selection and Enrichment Analysis

The variant is considered the “same” if the mutation is at the same genomic locus with the same alternative allele. SNVs or indels that occurred more than once in ADHD and had less than 1% frequency in controls were marked as recurrent variants of interest and moved forward in analysis if they had an MAF lower than 1% in the NHLBI 6500 exome sequencing project (6500 ESP) [10], 1000 Genome [11], and the genome aggregation database (gnomAD V2 for rare recurrent variants in the coding region and V3 for intronic/intergenic rare recurrent variants) [12,13] upon annotation. Rare recurrent variants that only occurred in ADHD and not in any controls were referenced as “zero-in-control rare recurrent variants”. Fisher exact tests and Chi-square tests were performed to test the difference for the given variant between ADHD and controls.

The enrichment analysis was based on DAVID (database for annotation, visualization, and integrated discovery) bioinformatics platform [14] and the adjusted *p*-value was based on using the Benjamini–Hochberg method. The impact of mutations were predicted and annotated by multiple tools, including SIFT [15], LRT [16], Polyphen2 [17], MutationTaster [18], MutationAssessor [19], FATHMM [20], RadialSVM [21], and clinical database such as ClinVar [22]. The number of intronic rare recurrent variants is huge; therefore, we emphasized intronic indels and the loci with multiple genomic interpretations (e.g., beside a gene’s intronic region, in another gene’s UTR, up/downstream, ncRNA regions, etc.). The current pathway databases, such as KEGG, do not include ncRNA in the pathways. In our analysis, variants in exonic regions of ncRNAs were annotated by the coding-genes in which the variants maps to their intronic, UTR, or up/downstream regions. Meta-analysis (combined Chi-square test) was applied when combining two ethnicities together in order to explore the significant SNVs that were enriched in both ethnicities.

## 3. Results

### 3.1. Novel Stop Codon/Frameshift Variants in Known ADHD Genes

**Figure 1:**
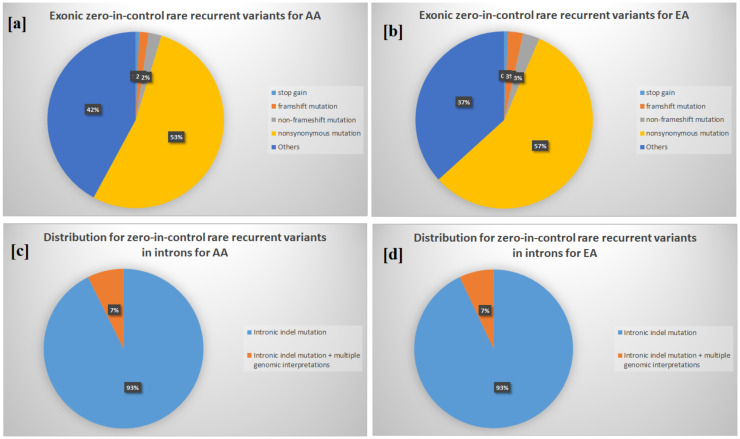
Distributions of rare recurrent variants. [**a**] exonic zero-in-control rare recurrent variants for African Americans (AA); [**b**] exonic zero-in-control rare recurrent variants for European Americans (EA); [**c**] intronic zero-in-control rare recurrent variants for AA; and [**d**] intronic zero-in-control rare recurrent variants for EA.

We identified 1815 zero-in-control rare recurrent variants in the AA cases and 1244 in the EA cases (Figure 1a,b, respectively). These include 12 stop-gain rare recurrent variants in AA and 9 in EA. Based on the ADHD database [23], none of the stop-gain mutations have been previously reported (Table 1). For AA, rare recurrent variants in *NUDT19* and *PCOLCE2* were predicted as deleterious by LRT and MutationTaster, meanwhile rare recurrent variants in *OR2D3*, *TAS2R14*, *POLN*, *ZNF880*, *ZNF433*, and *GLG1* were predicted as deleterious by MutationTaster. For EA, the variant rs371526758 (chr1: 10042426-G-A) in *NMNAT1* was predicted as deleterious by LRT and MutationTaster and reported as “pathogenic” in the ClinVar database. This variant resulted in a stop codon at exon 5 of *NMNAT1* in two EA ADHD patients. Previous studies show that mutations in *NMNAT1* cause Leber congenital amaurosis and white matter disorders [24]. In this regard, ADHD patients have been shown to have a delay in brain white matter development [25]. Another variant in the *FRY* gene was predicted to be deleterious by SIFT, LRT, and MutationTaster in EA.

Beside stop-gain mutations, there are 31 rare recurrent frameshift variants in AA and 34 in EA (Table 2). Multiple of these rare recurrent variants were found to impact ADHD associated or neurodevelopmentally related genes. For example, in AA, a novel frameshift insertion was uncovered at chr12:112036782, impacting the *ATXN2* gene. *ATXN2* is associated with multiple neurological diseases, and the variant is one of the top independent signal in our study. The frameshift insertion at the first exon of *ATXN2* is seen in two independent individuals. Six AA individuals contain rare recurrent frameshift variants at exon 3 and 62 for *ABCA13*, which were annotated as ADHD-related genes by Genecards [26] and is identified as overlapping gene for ADHD and Autism Spectrum Disorder (ASD) based on copy number variation analysis [27]. *DGKQ* is a reported gene in Parkinson’s disease [28], and five individuals have the frame insertion for *DGKQ* at exon 5. *FMNL2* has been identified in patients with depression [29] and is the top hit in a GWAS on schizophrenia patients in Japan [30]. For the EA ethnicity, six individuals have frameshift deletions at exon 9 for *CHD7*, the causative gene for CHARGE syndrome, where many patients also show ADHD behavior [31]. *HSD3B1* is related to the ADHD pathogenesis in rat [32] and a top GWAS hit for major depression [33]. *UNC5B* is an ADHD-associated GWAS locus [34] and contains frame insertion for two EA individuals. In the pathway enrichment analysis, 36 genes with stop-gain or frameshift mutations in AA show significant enrichment in the G-protein coupled receptor signaling pathway (adjusted *p*-value = 0.018).

### 3.2. Enrichment in Homophilic Cell Adhesion of Rare Recurrent Variants in Noncoding RNA/Introns

A total of 853 noncoding RNA (ncRNA) transcripts contain zero-in-control rare recurrent variants for AA and 581 for EA. The ncRNAs in AA show enrichment of specific disease classes, including METABOLIC (adjusted *p*-value = 8.7 × 10^−5^), DEVELOPMENTAL (adjusted *p*-value = 0.002), PSYCH (adjusted *p*-value = 0.013), and NEUROLOGICAL (adjusted *p*-value = 0.036) through DAVID, but not in any biological processes or pathways. In contrast, 581 ncRNAs in EA show significant enrichments in biological processes of homophilic cell adhesion via plasma membrane adhesion molecules (adjusted *p*-value = 1.9 × 10^−16^) and in calcium ion binding (adjusted *p*-value = 6.7 × 10^−10^). There are 1221 transcripts with intronic indels in EA, and they also show enrichment in homophilic cell adhesion via plasma membrane adhesion molecules (adjusted *p*-value = 2.8 × 10^−10^). Although the distributions of intronic rare recurrent variants are similar in AA and EA (Figure 1c,d), no enrichment was found for the AA group. Through datamining procedures, we found that the enrichment is due to a 4-bp intronic deletion at chr5:140806031, one of the protocadherins family genes (also found to be an exonic indel for the noncoding RNA HGB8P). A previous study suggested that epigenetic regulation of PCDHs in the human brain associated with brain disorders through cell–cell adhesion in the nervous system [35].

### 3.3. Differences between Two Ethnicities and Meta-Analysis

**Figure 2:**
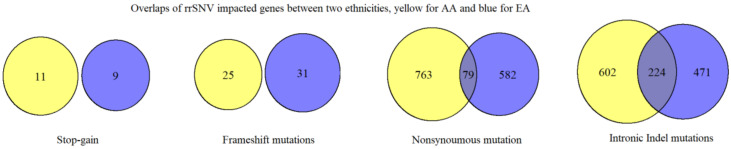
Numbers of overlaps for rare recurrent single-nucleotide variant (rrSNV)-impacted genes.

**Figure 3:**
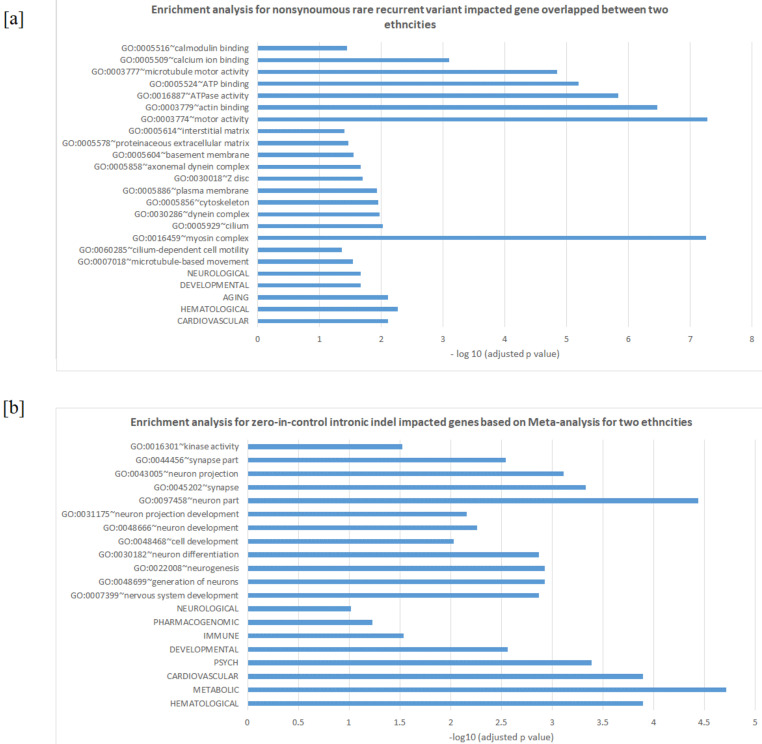
Enrichment analysis. (**a**) for nonsynoumous rare recurrent variant impacted gene overlapped between two ethnicities and (**b**) for zero-in-control intronic indel impacted genes based on Meta-analysis for two ethnicities. the x-axis is on the scale of −log10 (adjusted *p*-value) and the y-axis is in terms of enriched disease classes and gene ontology.

The overlap of genes impacted by zero-in-control rare recurrent variants is small between the two ethnicities, regardless of the mutation types (Figure 2). No overlap was identified for stop-gain or frameshift mutations, and only 79 genes that were impacted by nonsynonymous mutations are seen in both ethnicities. For genes impacted by nonsynonymous rare recurrent variants, 19 are ADHD-associated in AA and 14 in EA, with three of the ADHD-associated genes, *LRP1B, NRXN3,* and *SDK2*, present in both ethnicities (Table 3). No enrichment for pathways was identified for the overlapped genes based on nonsynonymous mutations and intronic indels. However, if we expand zero-in-control rare recurrent variants to rare recurrent variants (less than 1% frequency in controls), the number of overlapped gene increases to 703, shows significant enrichment in the myosin complex (adjusted *p*-value = 5.5 × 10^−8^), and reveals enrichment for disease classes such as neurological and developmental through DAVID (Figure 3a). Myosin is identified as risk factor for adult ADHD susceptibility and severity [36], and the gene *MYO16*, which contains rare recurrent variants in exon 15 for AA and exon 32 for EA, is a shared susceptibility gene between ADHD and ASD [37].

Meta-analysis was applied when combing the two ethnicities together as described in the Methods section. No exonic zero-in-control rare recurrent variants passed the threshold *p* < 0.05. On the other hand, there are 498 transcripts that contain zero-in-control rare recurrent intronic indels, and the corresponding genes are highly enriched in the disease classes METABOLIC, PSYCH, and NEUROLOGICAL and the Gene Ontology biological processes, such as neuron development and neuron differentiation (Figure 3b). There were 39 genes impacted by 47 zero-in-control rare recurrent variants that were identified in neuron development, neuron differentiation, and neurogenesis procedures (Table 4), and 8 of those genes (*NRXN3*, *DCDC2*, *NRXN1*, *MAGI2*, *CTNNA2*, *LINGO2*, *PRKG1*, and *CLASP2*) were identified in previous studies for ADHD [23]. These results suggest that noncoding indel mutations could potentially alter gene expressions in neuronal networks and could impact the biological process-related nervous system perturbations in ADHD.

### 3.4. Rare Recurrent Variants in Noncoding Regions of the Metabotropic Glutamate Receptor (Mglur) Pathway Genes

The glutamatergic neurotransmitter system plays an important role in ADHD, and the mGluR activator fasoracetam showed promising results when applied in clinical trials of adolescents harboring mutations in mGluR network genes [38]. Based on a meta-analysis for noncoding zero-in-control rare recurrent variants, we identified 7 novel intronic indels in 7 mGluR pathway genes, including DLGAP1, GNAQ, GNG2, GRIK2, GRM7, PRKCG, and PRKACB (Table 5). These rare recurrent variants have not been previously identified in esp6500 and the 1000 Genome database and have very low frequencies (<0.5%) in gnomAD.

## 4. Discussion

ADHD is a common, inheritable neurodevelopmental disorder largely of unknown etiology. Previous studies suggest that ADHD is more likely to be regulated and impacted at the level of biological pathways instead of an individual gene [2,39]. Rare copy number variation (CNV) has been reported in up to 25% of children with ADHD [4]. Our previous study in these subjects replicated multiple CNVs associated with ADHD from previous studies, and we identified clustering of noncoding structural variations (SVs) in neuroactive ligand–receptor interaction pathways [40].

Previous studies are highly focused on SNVs in coding regions; on the other hand, noncoding genomic structural variations and noncoding genes have been shown to play important roles in many human diseases, including other neurodevelopmental diseases such as autism and intellectual disability [41]. Another issue to consider is the ethnicity of ADHD patients, since previous studies have largely focused on Caucasians of European ancestry (EA) populations rather than studies in African American (AA) populations and other minority populations. In this study, we performed whole genome sequencing analysis in 205 ADHD patients, including 116 AA and 89 EA, using 670 non-ADHD control subjects from the Center for Applied Genomics at CHOP to explore the potential impact of rare recurrent SNVs on neuronal networks involved in the pathogenesis of ADHD and potential differences between AA and EA patients. 

We uncovered multiple novel rare recurrent variants in coding regions that present a plausible role in the pathogenesis of ADHD and further supported by their absence in population databases and non-ADHD controls. These include 12 vs. 9 stop-gain rare recurrent variants and 31 vs. 34 rare recurrent frameshifts variants in AA and EA patients, respectively. Two genes (*ATXN2* in AA and *UNC5B* in EA) have been reported as ADHD associated in previous studies [34,42]. No overlap was identified for rare recurrent frameshift variants between the two ethnicities (Figure 2). The portion of overlapping genes is also small for genes containing nonsynonymous rare recurrent variants (9% for AA and 12% for EA), with 2% of them classified as known ADHD-associated genes based on literature support. Exonic rare recurrent variants were observed in both of the ethnicities with minimum overlap, which suggests that exonic mutations impact AA and EA by distinct rare recurrent variants in different genes.

The most significant finding in this study is the impact of noncoding rare recurrent variants, especially intronic indels, on biological processes and pathways for ADHD. While intra-genomic indels may affect the transcriptional machineries (i.e., transcription initiation and enhancer interaction), these regulatory effects tend to be less severe than the functional effects of intronic indels by causing intron retention and loss-of-function [43]. The functional mechanisms of these intronic indels remain to be clarified as we did not identify any particular DNA functional elements changed by these indels by pattern recognition based on the previous study [44]. Unlike exonic rare recurrent variants, intronic indels in this study have much higher overlap (37% for AA and 48% for EA). No exonic rare recurrent variants were seen in meta-analysis for both ethnicities, but intronic indels were seen in loci corresponding to more than four hundred transcripts and 239 genes. These genes are highly enriched in biological processes involving neurogenesis, such as neuronal development (adjusted *p*-value = 5.5 × 10 ^−3^) and neuronal differentiation (adjusted *p*-value = 1.4 × 10 ^−3^). The neurogenesis-related genes have higher portion of known ADHD genes (18%), including *CTNNA2*, one of the top hits of ADHD GWAS studies associated with the metabotropic glutamate receptor (mGluR) pathway [4]. The 1-bp intronic indel deletion occurred at chr2: 80029140 in three AA and one EA patients. 

In two independent EA patients, we identified a four-base intronic deletion at chr5:140806031 covering 18 genes of the protocadherin (Pcdhs) families A and B, while the locus is also the exonic region for noncoding RNA, *HGB8P*. PCDHs are critical for the formation and function of neural circuits, including dendrite arborization, axon outgrowth and targeting, synaptogenesis, and synapse elimination, and epigenetic regulation of clustered PCDHs has been shown to play roles in brain developments. The molecular and functional interaction between protocadherin-γC5 and GABAA receptors was proven in rats [45]. GABAA has been shown to play a role in inhibitory neurons in children with ADHD [46], suggesting that there may be interplay between the intronic indels in PCDHs. Beside GABAA receptors, glutamate is another excitatory and inhibitory neurotransmitter in the brain, and glutamatergic gene variants disrupt mGluR neurotransmitter signaling. In a recent clinical trial, fasoracetam (NFC-1) treatment significantly improved symptoms in ADHD adolescents where rare recurrent variants impacting glutamatergic gene served as biomarkers [38]. We identified seven intronic indels in mGluR gene networks based on our meta-analysis, including a 10-bp insertion for *GRM7* at chr3:7515182. Another notable observation is that ADHD patients with a *CNTN4* mutation phenotype tend to be more severe, with a higher prevalence of emotional dysregulation and demonstrate enriched response to fasoracetam. We uncovered two nonsynonymous SNVs in *CNTN4*, one at chr3:3084045-A-T exon 21 in EA and another one at chr3:3030048-G-A exon 14 in AA, both predicted as deleterious based on prediction tools. Although no rare recurrent variants were identified in *CNTN4*, we found another gene, *CNTN6*, from the Contactins family that contains an intronic indel, a rare recurrent variant that passed the significant threshold of meta-analysis at chr3:1136572 (2-bp deletion).

In summary, we conducted a whole genome analysis in ADHD patients using whole genome sequencing data that takes noncoding regions and ethnicity factors into consideration. The results show that rare recurrent indels in noncoding regions, especially introns, may play an important role in the development and progression of ADHD, suggesting that WGS may be a powerful tool to discover novel molecular mechanisms of ADHD. Additionally, our study highlights that genomic-level population differences exist between European Caucasian and African American patients. This study focused on zero-in-controls rare variants. As a major weakness of this approach, the individual rare variants presented in this study lack statistical significance after correction for multiple tests due to their low frequencies. The individual rare recurrent variants presented in this study as compelling ADHD candidates warrant further study in larger-scale studies.

## Figures and Tables

**Table 1:** Novel stop-gain rare recurrent variants.

Ethnicity	avsnp147	chr	Locus	Ref	Alt	ADHD Occurrences	Genes	esp6500 Frequency	1000Genome Frequency	gnomAD Frequency
	rs369719214	19	33183093	G	A	2	NUDT19	6.0 × 10^−4^	6.0 × 10^−4^	6.0 × 10^−4^
	rs116059545	3	142537266	G	A	2	PCOLCE2	2.0 × 10^−4^	2.0 × 10^−4^	2.0 × 10^−4^
	rs146817618	11	6942649	T	A	2	OR2D3	2.0 × 10^−4^	.	3.0 × 10^−4^
	rs34789740	12	11091287	G	A	2	TAS2R14	1.4 × 10^−3^	4.0 × 10^−4^	8.0 × 10^−4^
	rs375920305	19	36259319	C	T	4	PROSER3	7.0 × 10^−4^	8.0 × 10^−4^	3.0 × 10^−4^
AA	.	19	52888074	-	ATCATGAGGTCAGGAGATCGAGACCATCCTGGCTAACAAGGTGAAACC	2	ZNF880	.	.	1.8 × 10^−3^
	rs150768729	4	2073958	C	T	2	POLN	2.0 × 10^−4^	.	3.3E-05
	rs370330395	19	52887191	C	T	3	ZNF880	1.3 × 10^−3^	6.0 × 10^−4^	1.2 × 10^−3^
	rs148053441	14	74361075	G	A	2	ZNF410	.	4.0 × 10^−4^	1.0 × 10^−3^
	rs181032032	19	12126454	G	A	2	ZNF433	5.0 × 10^−4^	4.0 × 10^−4^	7.0 × 10^−4^
	rs147487823	8	29202959	G	A	2	DUSP4	.	2.0 × 10^−4^	3.2 × 10^−5^
	rs138842904	16	74486025	G	A	2	GLG1	9.0 × 10^−4^	4.0 × 10^−4^	8.0 × 10^−4^
	rs147869298	4	140625183	C	T	2	MGST2	1.5 × 10^−3^	6.0 × 10^−4^	5.0 × 10^−4^
	.	9	104239264	-	ATTAAAAA	2	TMEM246	.	.	7.0 × 10^−5^
	rs145322761	14	96730863	C	T	2	BDKRB1	2.8 × 10^−3^	1.4 × 10^−3^	2.0 × 10^−3^
	rs138652787	7	23871861	C	G	2	STK31	1.8 × 10^−3^	6.0 × 10^−4^	1.6 × 10^−3^
EA	.	2	11925167	-	ATA	3	LPIN1	.	.	0.0E+00
	rs142358325	9	140139138	G	A	2	FAM166A	5.0 × 10^−4^	4.0 × 10^−4^	3.0 × 10^−4^
	.	13	32731436	C	T	2	FRY	.	.	.
	rs370788593	10	82348410	C	T	2	SH2D4B	7.7 × 10^−5^	.	3.2 × 10^−5^
	rs371526758	1	10042426	G	A	2	NMNAT1	2.0 × 10^−4^	.	6.5 × 10^−5^

**Table 2:** Selected rare recurrent frameshift variants.

Ethnicity	chr	Locus	Ref	Alt	ADHD Occurrences	Genes	esp6500 Frequency	1000Genome Frequency	gnomAD Frequency
	7	48237838	TTTG	-	2	ABCA13	.	.	3.38 × 10^−5^
	7	48237845	-	GA	2	ABCA13	.	.	0
	7	48237846	-	CA	2	ABCA13	.	.	0
AA	12	112036782	-	GCTGCTGCTGCTGC	2	ATXN2	.	.	.
	4	962079	-	TGCCTCTCCTGCCCCGCCCCCCCAACTCCTC	3	DGKQ	.	.	0.0021
	4	962079	-	TGCCTCTCCTGCCCCGCCC	2	DGKQ	.	.	0.0014
	2	153417444	-	GCCGT	2	FMNL2	.	.	0
	2	153417451	GCCCTGG	-	2	FMNL2	.	.	.
	8	61732577	GCTTT	-	3	CHD7	.	.	7.63 × 10^−5^
	8	61732592	TT	-	3	CHD7	.	.	6.53 × 10^−5^
EA	1	120056817	-	G	2	HSD3B1	.	.	0.0005
	1	120056818	-	AA	2	HSD3B1	0.0007	.	0.0005
	10	73044507	-	G	2	UNC5B	.	.	0.0004

**Table 3:** Nonsynonymous zero-in-control rare recurrent SNV variants (rrSNV) with ADHD association from previous studies.

Ethnicity	chr	Locus	Ref	Alt	avsnp147	ADHD Occurrences	Genes	SIFT	Polyphen2	LRT	Mutation Taster	Mutation Assessor	FATHMM	Radial SVM
	8	26721808	A	G	rs201951243	2	*ADRA1A*	D	D	N	D	L	T	T
	10	135139570	C	T	rs149764782	2	*CALY*	T	P	N	D	M	.	T
	10	73501613	C	T	rs200664666	2	*CDH23*	T	D	D	D	M	.	T
	2	141459361	G	A	rs147598746	2	*LRP1B*	T	B	N	N	N	D	T
	18	47429043	C	A	rs114221227	2	*MYO5B*	D	D	D	D	M	T	T
	14	80327577	A	G	.	2	*NRXN3*	.	B	.	D	.	T	T
	2	42991127	G	A	rs146033252	2	*OXER1*	T	B	.	N	N	T	T
EA	17	71410819	G	T	rs143251430	2	*SDK2*	D	P	D	D	L	T	T
	1	177930009	C	T	rs185756583	2	*SEC16B*	D	D	D	D	M	T	T
	10	98945389	G	A	rs375276519	2	*SLIT1*	D	D	N	N	N	T	T
	6	24658948	T	C	rs61757564	3	*TDP2*	T	B	N	N	L	T	T
	11	78383209	A	T	rs201179027	2	*TENM4*	.	D	D	D	L	T	D
	11	78369726	C	T	rs199594129	2	*TENM4*	.	D	N	D	N	D	T
	3	36873195	C	T	rs373979668	2	*TRANK1*	D	B	D	N	M	T	T
	19	57764649	G	A	rs377720732	2	*ZNF805*	D	B	.	N	L	T	T
	11	74988453	C	T	rs370469526	2	*ARRB1*	T	B	D	D	N	T	T
	4	96025649	C	G	rs145700191	2	*BMPR1B*	T	B	N	D	L	T	D
	9	90585782	C	T	rs28364955	2	*CDK20*	T	P	D	N	L	T	T
	7	50566868	C	T	rs573103547	2	*DDC*	D	D	D	D	M	T	T
	9	1056887	C	T	rs147461872	2	*DMRT2*	D	D	U	D	M	T	T
	2	141250262	T	C	rs140458851	2	*LRP1B*	T	B	N	D	N	D	T
	7	77648999	C	A	rs773082728	2	*MAGI2*	T	B	.	D	N	T	T
	15	91455385	G	A	rs150171248	2	*MAN2A2*	T	B	D	D	M	D	T
AA	1	11856376	C	T	rs150847674	2	*MTHFR*	T	P	D	D	M	D	D
	12	117768154	C	T	rs76090928	2	*NOS1*	D	B	D	D	L	T	T
	14	79454391	T	G	.	2	*NRXN3*	.	D	D	D	.	T	T
	20	4773214	G	A	rs148303159	2	*RASSF2*	T	B	N	N	L	T	T
	17	71397825	T	C	rs138152327	2	*SDK2*	T	B	N	N	M	T	T
	6	155577706	C	A	rs114296676	2	*TIAM2*	T	D	D	N	M	T	T
	5	14487903	C	T	rs533386148	3	*TRIO*	T	P	N	D	N	T	T
	1	55571832	C	T	.	2	*USP24*	T	P	D	D	L	T	T
	19	58772917	A	C	rs148565349	2	*ZNF544*	T	B	.	N	L	T	T
	5	123982926	T	C	rs149444271	2	*ZNF608*	T	B	D	D	M	T	T
	5	123980164	T	C	rs113873110	2	*ZNF608*	.	B	N	D	L	T	T

**Table 4:** Intronic indel rare recurrent SNV (rrSNV) that impacted the genes identified in neuron development and neuron differentiation procedures.

ADHD Associated Genes	Gene	chr	Locus	Ref	Alt	avsnp147	ADHD AA Occurrences	ADHD EA Occurrences	meta *p*-Value
Y	*MAGI2*	7	77687294	-	TATA	.	4	1	0.008
-	*ZMYND8*	20	45929674	-	TGTGTGTA	.	4	1	0.008
-	*ERBB4*	2	212606179	-	ACACACAC	.	4	1	0.008
-	*SOX6,*	11	16031671	-	ACACACAC	.	4	1	0.008
-	*ALCAM*	3	105241984	-	AA	.	3	2	0.009
-	*BRINP3*	1	190425195	A	-	.	1	4	0.015
Y	*NRXN3*	14	78954086	ATAAATAAATAAATAA	-	.	2	3	0.012
-	*COL25A1*	4	110139513	-	T	rs34056401	2	3	0.012
-	*CBFA2T2*	20	32229919	TTTTTGTGTGTGTG	-	.	2	3	0.012
Y	*DCDC2*	6	24280004	T	-	rs563616388	1	4	0.015
Y	*DCDC2*	6	24256576	TA	-	rs556522905	1	4	0.015
Y	*NRXN1*	2	50393425	-	TG	.	1	4	0.015
Y	*NRXN3*	14	78937634	-	AC	.	3	1	0.038
Y	*CTNNA2*	2	80029140	T	-	.	3	1	0.038
-	*RORA*	15	61494733	T	-	.	3	1	0.038
-	*ARHGEF2*	1	155941370	-	AAAAAAAAAAA	.	3	1	0.038
-	*BICDL1*	12	120451243	-	GTGTGTGTGTGTGT	.	3	1	0.038
-	*PARD3*	10	34408932	-	TTTTTTTTTTT	.	3	1	0.038
-	*CNTN6*	3	1136572	AT	-	rs367911099	3	1	0.038
-	*DAB1*	1	58589583	AC	-	.	3	1	0.038
-	*COL25A1*	4	109743199	-	TTTTTTTT	.	3	1	0.038
-	*ANKS1A*	6	34891869	-	GTGT	.	3	1	0.038
-	*PTPRD*	9	9632617	A	-	.	3	1	0.038
-	*NPTN*	15	73897440	-	GG	.	3	1	0.038
-	*SLC4A7*	3	27480018	-	AAAAT	rs141000029	3	1	0.038
-	*LAMB1*	7	107575792	-	TTTTTTTTTTTTTT	.	3	1	0.038
-	*DAB1*	1	58222892	-	TTTTTTTTTTTTTTTTTTTTTTTTTG	.	3	1	0.038
-	*RORA*	15	61494730	-	G	.	3	1	0.038
-	*GNAQ*	9	80366732	AAAA	-	.	3	1	0.038
-	*MTOR*	1	11178676	-	T	.	3	1	0.038
-	*DAB1*	1	58616263	AA	-	.	3	1	0.038
-	*LRP6*	12	12392929	-	A	.	3	1	0.038
-	*NCAM2*	21	22444188	-	TATCTAT	.	3	1	0.038
-	*SPTBN4*	19	41005988	-	AAAAAAAAAAAAAAAAAAAAAAAAAAAAAAGAAAAGAAAAATCTTTGCTGAGCATGGTGGTACAC	.	3	1	0.038
-	*RAB10*	2	26331748	-	TATT	.	3	1	0.038
Y	*CLASP2*	3	33577416	A	-	.	3	1	0.038
Y	*LINGO2*	9	28664998	-	ATATATAT	.	2	2	0.046
Y	*PRKG1*	10	52873716	-	GT	.	2	2	0.046
-	*TENM2*	5	166755213	-	T	.	2	2	0.046
-	*DNER*	2	230419651	GAGAGAAAAGGGAAGGG	-	.	2	2	0.046
-	*EXT1*	8	119053913	GAAG	-	.	2	2	0.046
-	*PTPRD*	9	8632122	TG	-	rs755867249	2	2	0.046
-	*ABL2*	1	179169177	T	-	.	2	2	0.046
-	*NOTCH3*	19	15280017	-	TCTCTCTC	.	2	2	0.046
Y	*NRXN1*	2	50932188	ACAC	-	.	2	2	0.046
-	*UST*	6	149379481	ATGTGTGTGT	-	.	2	2	0.046
-	*GCM1*	6	53006798	-	GAAA	.	2	2	0.046

**Table 5:** Rare recurrent variants in noncoding regions for mGluR genes.

Gene	chr	Locus	Ref	Alt	ADHD AA Occurrences	ADHD EA Occurrences	Meta-Analysis *p*-Value	esp6500 freq	1000Genome freq	gnomAD freq
*GRM7*	3	7515182	-	GAGAGAGAGA	2	3	0.012	.	.	.
*DLGAP1*	18	4330027	AT	-	3	1	0.038	.	.	0.0008
*GNG2*	14	52368681	-	A	3	1	0.038	.	.	.
*GRIK2*	6	102004196	-	TTTTTTTTTTTTTTTTTTTTTTTTTTTTTTTTTTTTTTTTTTTTTTTTT	3	1	0.038	.	.	.
*PRKACB*	1	84664799	-	ATAT	3	1	0.038	.	.	0.0038
*GNAQ*	9	80366732	AAAA	-	3	1	0.038	.	.	0.0006
*PRKCG*	19	54406144	-	AAAAAAAAAAAAAAAA	3	1	0.038	.	.	0.0022

## Data Availability

The data is available at the database of Genotypes and Phenotypes (dbGaP) portal with the accession number phs001165.
